# Hepatomegaly and Splenomegaly: An Approach to the Diagnosis of Lysosomal Storage Diseases

**DOI:** 10.3390/jcm13051465

**Published:** 2024-03-02

**Authors:** Teodoro Jerves Serrano, Jessica Gold, James A. Cooper, Heather J. Church, Karen L. Tylee, Hoi Yee Wu, Sun Young Kim, Karolina M. Stepien

**Affiliations:** 1 Department of Genetics, Yale School of Medicine, New Haven, CT 06510, USA; 2 Division of Genetics, Department of Pediatrics, Children’s Hospital of Philadelphia, Philadelphia, PA 19104, USA; goldj@chop.edu; 3 Willink Biochemical Genetics Laboratory, St Mary’s Hospital, Manchester University NHS Foundation Trust, Manchester M13 9WL, UK; james.cooper@mft.nhs.uk (J.A.C.); heather.church@mft.nhs.uk (H.J.C.); karen.tylee@mft.nhs.uk (K.L.T.); hoiyee.wu@mft.nhs.uk (H.Y.W.); 4 Division of Human Genetics, Department of Pediatrics, Cincinnati Children’s Hospital Medical Center, University of Cincinnati, Cincinnati, OH 45219, USA; sunyoung.kim@cchmc.org; 5 Salford Royal Organization, Northern Care Alliance NHS Foundation Trust, Adult Inherited Metabolic Diseases Department, Salford M6 8HD, UK; 6 Division of Cardiovascular Sciences, University of Manchester, Manchester M13 9PL, UK

**Keywords:** lysosomal storage diseases, hepatomegaly, splenomegaly, organomegaly, hepatosplenomegaly, biomarkers

## Abstract

Clinical findings of hepatomegaly and splenomegaly, the abnormal enlargement of the liver and spleen, respectively, should prompt a broad differential diagnosis that includes metabolic, congestive, neoplastic, infectious, toxic, and inflammatory conditions. Among the metabolic diseases, lysosomal storage diseases (LSDs) are a group of rare and ultrarare conditions with a collective incidence of 1 in 5000 live births. LSDs are caused by genetic variants affecting the lysosomal enzymes, transporters, or integral membrane proteins. As a result, abnormal metabolites accumulate in the organelle, leading to dysfunction. Therapeutic advances, including early diagnosis and disease-targeted management, have improved the life expectancy and quality of life of people affected by certain LSDs. To access these new interventions, LSDs must be considered in patients presenting with hepatomegaly and splenomegaly throughout the lifespan. This review article navigates the diagnostic approach for individuals with hepatosplenomegaly particularly focusing on LSDs. We provide hints in the history, physical exam, laboratories, and imaging that may identify LSDs. Additionally, we discuss molecular testing, arguably the preferred confirmatory test (over biopsy), accompanied by enzymatic testing when feasible.

## 1. Background

Hepatomegaly and splenomegaly (HSM) are the abnormal enlargement of the liver and spleen, respectively, which can be recognized by means of a physical exam or distinct imaging modalities [1,2]. Physical examination often identifies hepatomegaly and/or splenomegaly, yet palpation can miss, overestimate, or underestimate these findings [2,3]. Hence, imaging should follow an abnormal physical exam to confirm the organomegaly and obtain more details, such as the presence of liver or spleen cysts, masses or features suggestive of congestion or infiltrative disease. Currently, magnetic resonance imaging and computed tomography are considered the gold standard to determine liver and spleen volumes. Nevertheless, costs, radiation, claustrophobia, and metal prosthetics preclude their use as the first approach [4]. In those circumstances, ultrasound is affordable, readily available, and well tolerated [2,4].

The causes of both hepatomegaly and splenomegaly, separately or together, are numerous and include metabolic, congestive, neoplastic, infectious, toxic, or inflammatory conditions. The evaluation of HSM frequently entails an extensive work-up to address this broad differential diagnosis [5,6]. A HSM workup may be initiated by pediatricians, internists, gastroenterologists, and hematologists. At a minimum, the initial basic laboratory investigations may unveil abnormalities, i.e., hepatocellular damage, cholestasis, and synthetic liver dysfunction [7]. Thus, an organized, methodical approach to HSM is necessary to prevent invasive testing and decrease time to diagnosis.

Among those metabolic conditions in the differential diagnosis are lysosomal storage diseases (LSDs). These disorders are usually the purview of medical and biochemical geneticists [8,9]. LSDs are individually rare or ultrarare inherited metabolic diseases (IMDs), but as a group occur commonly with an approximate collective incidence of 1 in 5000 live births [10]. Most LSDs are inherited in an autosomal recessive manner, with the exceptions of mucopolysaccharidosis type II, Fabry disease, and Danon disease, which are inherited in an X-linked pattern. Lysosomes are organelles required for lipid, protein, and carbohydrate degradation, as well as signal transduction and ion homeostasis. Defects in lysosomal enzyme activity, integral membrane proteins, activators, and transporters lead to the accumulation of substrates and metabolites inside the organelle, impairing its function [11]. 

LSDs are heterogeneous conditions affecting multiple organs from the fetal period to adulthood with a broad spectrum of severity and onset of clinical manifestations. Attenuated forms of LSDs are now frequently recognized in adulthood. Referrals of adult patients with HSM to adult metabolic centers globally has increased exponentially in the last few years [12]. In such cases, screening for Gaucher disease, Niemann-Pick disease type C (NPC), cholesterol esterase storage disease (CESD) and acid sphingomyelinase deficiency (ASMD) are considered. 

Multidisciplinary management along with advanced therapeutics have improved mortality, morbidity, and quality of life for many individuals with LSDs. However, early diagnosis and management are essential to achieve these benefits. Without such interventions, the outcomes for many individuals with LSDs remain poor. Regardless of the prognosis, a definitive diagnosis can help identify other family members affected and provide guidance for family planning [13]. 

Once the diagnosis is established, appropriate referrals must be completed for the multidisciplinary management and surveillance of the particular LSD. Disease-specific therapies are commercially available for some LSDs and remain investigational for others. Enzyme replacement therapy (ERT) is currently available for ASMD [14]. Therapeutic approaches, apart from ERT for Gaucher disease, include substrate reduction therapy [15], and advanced liver disease invariably requires liver transplantation [16]. Patients with NPC are primarily managed with supportive treatment and multidisciplinary care. There is no curative therapy, but miglustat, as a disease-modifying therapy, has been shown to be more effective in patients with late-onset neurological symptoms [17]. Hematopoietic stem cell transplantation (HSCT) is a therapeutic option previously trialled in several LSDs with neurocognitive dysfunction including Hurler syndrome [18].

This review article navigates the diagnostic approach for individuals with HSM, particularly focusing on LSDs. We provide hints in the history, physical exam, laboratories (basic, biomarkers, and biochemical urine tests), and imaging to identify those cases that are more likely to be one of the LSDs. Additionally, we discuss molecular testing, accompanied by enzymatic testing when feasible. Contrarily, biopsies are invasive and should be the last resource for the diagnosis.

## 2. Causes of Hepatomegaly and/or Splenomegaly

### 2.1. Common Causes 

Common causes are investigated first unless there is remarkable evidence concerning one or more LSDs, and further details are discussed in the sections below. Among these common causes of HSM are metabolic (Hemochromatosis, Wilson’s disease), congestive (heart failure, thrombosis), neoplastic (leukemia, lymphoma, hepatoblastoma, hepatocellular carcinoma and secondary metastasis to liver), hematologic (thalassemia, sickle cell anemia), infectious (cytomegalovirus, toxoplasmosis, hepatitis virus), inflammatory (sarcoidosis, systemic lupus erythematosus), toxic (acetaminophen overdose), and infiltrative (amyloidosis) causes [19,20]. 

### 2.2. LSDs

LSDs usually present with non-tender hepatomegaly and/or splenomegaly, as well as smooth liver and spleen borders [21], but it is important to emphasize that an unsmooth liver/spleen does not exclude LSDs. HSM is more common in the severe and early-onset forms of LSDs. Table 1 presents a comprehensive list of all the LSDs with HSM as a clinical manifestation, grouped into classes and coupled with their respective genes. Disorders of lysosome-related organelles, such as Chediak–Higashi and Hermansky–Pudlak syndromes, are excluded from this review, even though they could potentially lead to HSM in the setting of hemophagocytic lymphohistiocytosis [22].

HSM is a common feature of mucopolysaccharidoses (MPSs) type I-VII, ASMD, lysosomal acid lipase deficiency (Wolman disease in children or CESD in adults), GM1 gangliosidosis type I, mucolipidosis type II, galactosialidosis, saposin C deficiency, NPC, and Gaucher disease. It is rare in other LSDs such as aspartylglucosaminuria and Sandhoff, whereas, it is absent in metachromatic leukodystrophy, among others. 

### 2.3. Other Genetic Diseases

Other genetic diseases can present with splenomegaly, like sickle cell disease or hepatomegaly in the case of alpha-1-antitrypsin deficiency and Beckwith–Wiedemann syndrome [21,52]. Aside from LSDs, other IMDs that lead to HSM are urea cycle disorders, classical galactosemia, glycogen storage disorders, hereditary fructose intolerance, tyrosinemia type I, prolidase deficiency, mitochondrial diseases, fatty acid oxidation defects, congenital disorders of glycosylation, peroxisomal disorders, among others [19]. Thus, genetic disorders and IMDs are important to include in a differential diagnosis for HSM.

## 3. Diagnostic Work-Up for LSDs

The initial work up for HSM should include imaging and basic laboratories that evaluate common etiologies. When LSDs are considered in the differential diagnosis, the patient should preferably be referred to a biochemical geneticist or any other specialist familiar with these conditions. However, due to workforce and geographic limitations, initial evaluation using genetics is not always possible. Thus, primary care providers should be familiar with common presentations of LSDs. In the following paragraphs, we describe hints in the history and physical exam that increase the suspicion of LSDs in the setting of HSM [53,54]. A concern for LSDs or the absence of the definitive diagnosis after testing for the common causes should trigger an expedited referral to work-up LSDs and other genetic diseases.

**Figure 1:**
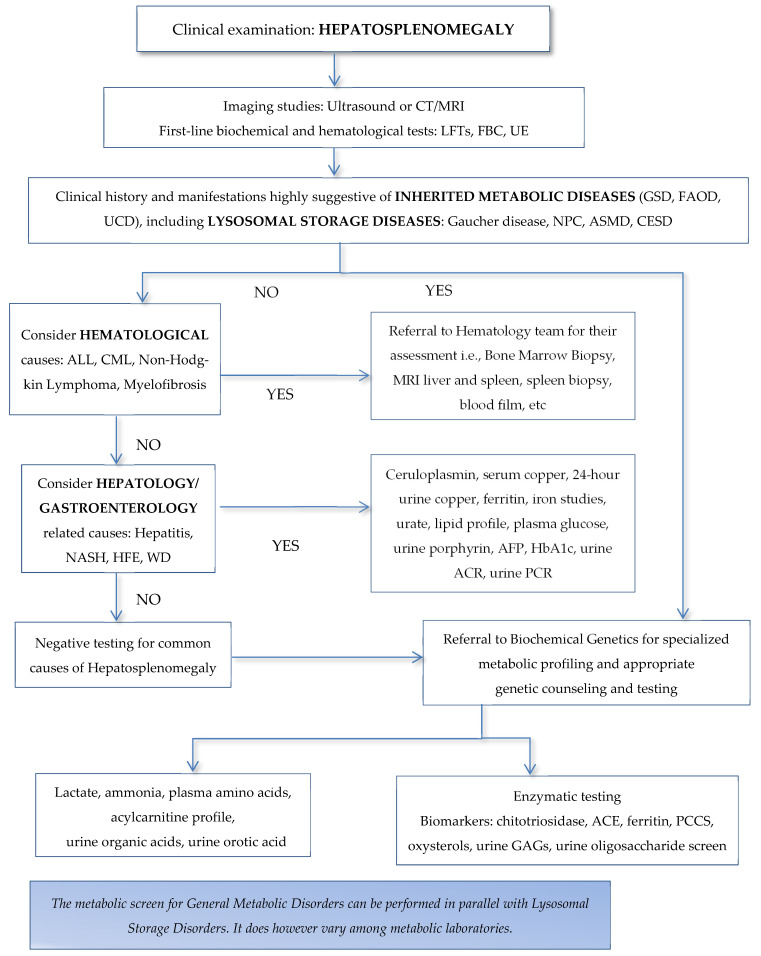
A diagnostic algorithm for hepatosplenomegaly. ACE = angiotensin converting enzyme; AFP = alpha-fetoprotein; ASMD = acid sphingomyelinase deficiency; CESD = cholesterol esterase storage disease; CML = chronic myeloid leukemia; FBC = full blood count; FAOD = fatty acid oxidation disorders; GAGs = glycosaminoglycans; GSD= glycogen storage diseases; HFE = Hemochromatosis; LFTs = liver function tests; PPCS = N-palmitoyl-O-phosphocholineserine; NASH = non-alcoholic steatohepatitis; NPC = Niemann–Pick disease type C; UCD = urea cycle disorders; UE = urea and electrolytes; WD = Wilson disease; ACR = albumin/creatine ratio; PCR = protein to creatine ratio.

The diagnostic algorithm of HSM in adult patients is outlined in Figure 1. Consanguinity increases the chances of IMDs and LSDs, and most of these conditions are caused by biallelic pathogenetic variants in the correspondent genes [55]. LSDs may be recognized during the perinatal period with non-immune hydrops and HSM detected by a prenatal ultrasound [53]. Intellectual disability, developmental delay, developmental regression, and autism are signs present during childhood, adolescence, and adulthood in many LSDs [56,57,58]. Similarly, behavioral and mental health problems, namely psychosis, aggressiveness, cognitive decline, and early-onset dementia, increase the suspicion of late-onset LSDs in adults [54], mainly NPC [59]. 

### 3.1. Clinical Manifestations of LSDs

Clinical manifestations of LSDs: MPSs, oligosaccharidoses and mucolipidoses have findings of a particular form of skeletal dysplasia called “dysostosis multiplex”. Coarse facial features that become more prominent with age may be an important diagnostic sign [39,60]. Coarse facial features involve the presence of frontal bossing, a depressed nasal bridge, an enlarged tongue, prominent supraorbital ridges, rounded cheeks, thick lips, and wide spaced teeth. Additionally, progressive macrocephaly is noticed in children with GM1 gangliosidosis, Sandhoff diseases and GM2 gangliosidosis due to GM2 activator protein deficiency [61]. Contrary to childhood-onset LSDs, attenuated forms of these conditions may present subtly without any striking facial or bony features that are usually characteristic for LSDs. Other systems that may be affected include dermatological findings. Angiokeratomas are present in certain oligosaccharidoses [39] and abundant dermal melanocytosis is common in children with MPSs [62,63]. However, other skin disorders have also been observed in adults with MPS disorders [64]. Farber disease has a classic triad that encompasses a hoarse voice, subcutaneous nodules, and joint deformities. Attenuated forms, often mimicking juvenile arthritis, have previously been described [26]. Individuals with Sialidosis and Galactosialidosis can develop nephrotic syndrome, while renal Fanconi syndrome is common in cystinosis [65,66,67].

In the presence of HSM, further evaluations recommended an ophthalmological exam that may reveal a cherry-red spot concurrent with severe early-onset forms of LSDs, whilst corneal clouding and corneal cystine crystals can be present in children or adults [67,68,69]. On the other hand, vertical supranuclear gaze palsy is a key clinical feature of NPC, and the slowing of horizontal ocular saccades is characteristic of neuronopathic Gaucher disease [70,71]. 

### 3.2. Imaging

Imaging of the liver and spleen can be helpful in ruling out benign and malignant neoplasms along with etiologies that produce cysts [20] or gaucheromas [72]. Abdominal imaging may also detect disease-specific pathologies, such as adrenal calcifications in Wolman disease [73].

The application of imaging modalities other than MRI or ultrasound of the liver has been evaluated in LSDs. As an example, elastography has been shown to be useful in the assessment of liver fibrosis in Gaucher disease [74]. The additional value of the fibroscan is the non-invasive evaluation of controlled attenuation parameters and liver stiffness [75]. 

The skeletal survey can identify dysostosis multiplex that includes findings like J-shaped sella, the thickening of diploic spaces, short and thick clavicles, broad oar-shaped ribs, dysplastic vertebral bodies, scoliosis, inferior pelvic tapering, rounded iliac wings, hip dysplasia, shortened long bones with hypoplastic epiphyses, short and wide metacarpals with thin cortices and proximal pointing, irregular and hypoplastic carpal and tarsal bones. Dysostosis multiplex is characteristic of MPSs, oligosaccharidoses and mucolipidoses [39,60,64]. Bone infarction, arthropathy, and lytic lesions are classical features of Gaucher disease [76], while reduced bone mineral density is a feature of Gaucher disease and ASMD, especially in adult patients [77].

### 3.3. First-Line Laboratories

First-line laboratories involve a liberal workup which is required to determine additional liver problems such as hepatocellular damage, cholestasis, and cirrhosis. Most of the LSDs that present with organomegaly have normal liver function test results; nonetheless, the absence of such laboratory abnormalities does not rule out these conditions [24]. Cholestasis is commonly observed in early-onset NPC; liver failure can be evidenced in ASMD and NPC; elevated liver function test results can be expected in Farber and Pompe disease; rare cases of cirrhosis have been reported in Gaucher disease [78], as well as MPS type I and II [9]. CESD is suspected in individuals with high cholesterol, low-density lipoprotein, and triglycerides [79], who may also have persistent mildly elevated liver function tests. The childhood-onset form, Wolman disease, manifests with failure to thrive and very abnormal biochemical and hematological tests. 

Apart from biochemical blood and urine tests, hematological investigations are important. Chronic anemia, mild to severe thrombocytopenia and abnormal coagulation tests are often seen in pediatric and adult-onset (attenuated) cases of Gaucher disease. Mild but persistent thrombocytopenia of around 110,000 platelets per microliter was reported as the only laboratory investigation in some attenuated Gaucher disease patients, which led to further investigations and diagnosis at the age of 35 (personal observations of KMS). A range of cytological abnormalities have been described in some LSDs, especially vacuolated lymphocytes [80] and azurophilic inclusions in lymphocytes [81].

Certain non-LSD metabolic disorders may have similar hepatic manifestations and should be considered as outlined above in Section 2.3. Wilson disease and alpha-1-antitrypsin deficiency are likely if cirrhosis is also present. Hepatomegaly in conjunction with hypoglycemia and seizures can be associated with hepatic glycogen storage diseases or gluconeogenesis defects; tyrosinemia type I or Fanconi–Bickel disease should be considered with co-existing renal tubulopathy; and hereditary fructose intolerance often demonstrates abnormal liver function. These disorders are diagnosed mainly in children, but should also be included in the differential diagnoses in adulthood, especially if temporally related to a catabolic stressor like fasting, pregnancy, or steroid use [7].

First line basic and metabolic investigations for HSM are:

- Liver function tests: ALT, AST, GGT, alkaline phosphatase, bilirubin, albumin, alpha-fetoprotein, total bile acids
- Full blood count, coagulation profiles, peripheral blood smears
- Renal function tests, electrolytes, and blood gases: urea, creatinine, sodium, potassium, bicarbonate, chloride, calcium, phosphate, uric acid
- Lipid profile including triglycerides, cholesterol
- Glucose
- Lactate
- Ammonia
- Alpha-1-antitrypsin
- Copper and Ceruloplasmin
- Ferritin and iron studies
- Free and total carnitine and acylcarnitine profile
- Plasma and urine amino acids
- Urine organic acids
- Urine ketones
- Reducing substances urine test

Second-line laboratory investigations aiming at the diagnosis of LSDs include analyses of enzyme function, glycosaminoglycans (GAGs), oligosaccharides, and other specific and non-specific biomarkers. These analyses are performed in specialized biochemical laboratories and are not readily available in general commercial labs.

### 3.4. Genetic Testing

Genetic testing is a useful non-invasive (blood, saliva and buccal swab samples are commonly used) diagnostic approach in centers with and without limited access to the specialized LSD biochemical diagnostic services because it can capture genetic variants (formerly known as mutations) associated with LSDs, as well as other genetic diseases concurrently. Pre-and post-test genetic counseling is highly encouraged. Molecular testing can be diagnostic when likely pathogenic or pathogenic variants are identified. Nevertheless, findings like variants of uncertain clinical significance can lead to more targeted/specific biochemical tests including enzymology or biomarker evaluations that eventually confirm the diagnosis and may upgrade variant classification. Variants of uncertain clinical significance are non-diagnostic and are reported more commonly in populations underrepresented in the genomic databases. Genetic testing frequently occurs in tandem with or after the laboratories discussed below: biochemical urine tests, enzymatic assays, and biomarker evaluation.

Today, Sanger sequencing for single gene testing is rarely used. In contrary, short read next-generation sequencing has become widely available and more affordable, facilitating different gene testing approaches ranging from gene panels to exome or genome sequencing [82]. Gene panels simultaneously investigative multiple genes associated with a specific clinical presentation or group of diseases. For instance, gene panels are commercially available for LSDs or HSM [83]. It is important to check whether or not the genes of interest are included in the ordered genetic testing and the estimated turnaround time (about 3–4 weeks for most commercial laboratories). In the outpatient setting, gene panels or exome sequencing are the preferred option. Rapid exome or genome sequencing can deliver quicker results in emergency cases and the critical care setting within 1 week [84]. 

Likewise, some limitations must be considered. Depending on the type of genetic testing, some variants cannot be easily identified. As an example, gene panels based exclusively on short reads detected by massive-parallel sequencing might miss the deletion of a partial exon [85]. Moreover, pseudogenes can hinder the sequencing of specific genes, such as *GBA* and *IDS*, which are associated with Gaucher disease and MPS II, respectively [82].

### 3.5. Enzymatic Analyses

Enzymatic analyses on plasma, leukocytes or fibroblasts often can provide a definite biochemical diagnosis of LSDs. However, enzymatic testing is not available for disorders of integral membrane proteins. About 60% of lysosomal disorders are associated with a deficiency of a specific lysosomal hydrolase, and these account for approximately 85–90% of patients diagnosed [10]. Some laboratories routinely offer large panels while others are more selective. It is important for the clinical team to understand the diagnostic strategy of the referral laboratory to ensure that the requested panel includes the suspected disorders. Enzyme activities are routinely measured in plasma and leukocytes isolated from peripheral blood. The use of dried blood spots as an initial sample source has increasingly been developed for some enzymes [86].

Historically, enzyme activity was measured using cultured skin fibroblasts as a sample source, which have largely been replaced by using peripheral blood, but can still be required to establish a diagnosis in some atypical cases. The disadvantage of using fibroblasts is that it requires a skin biopsy, an invasive procedure. There is also a time delay for the fibroblast cultures to establish and grow. However, once the cultures have been established, they can be cryopreserved and recovered if further investigations are required. Cultured cells (usually fibroblasts) are the gold standard sample type to measure neuraminidase for the diagnosis of sailidosis and galactosialidosis, and they can be used for filipin staining in suspected cases of NPC [57,87,88].

When a potential biochemical diagnosis is identified by the deficiency of a sulfatase enzyme, further investigations are required to establish or exclude the differential diagnosis of multiple sulfatase deficiency (MSD). It has been found that not all sulfatase enzymes are deficient in MSD, particularly in attenuated patients. It is therefore recommended that at least three different sulfatase enzymes are measured [30,89]. Mucolipidoses are characterized by an increased enzymatic level of hydrolases in the plasma contrasted with the decreased level in the fibroblasts [45].

A widely described pitfall of lysosomal enzymatic analysis is pseudodeficiency. The enzyme activity is reduced in vitro, but individuals do not have the typical expected clinical manifestations of associated LSD [45]. Pseudodeficiency is explained by decreased specificity toward an artificial substrate used in assays due to the presence of pseudodeficiency variants. Enzyme activity can be reduced to levels that overlap with those seen in affected individuals. Additional biomarkers and genetic analysis may be required to clarify a potential diagnosis. Another rare complication of enzyme analysis can be normal enzyme activity towards artificial substrates, giving a normal result in an affected patient. This has been reported as an issue in some patients affected by ASMD with a specific variant *SMPD1*, p.Q294K (hg38) [90].

Importantly, the enzyme activity may differ in early-onset versus adult-onset cases. Although the correlation is not perfect, severe and early-onset LSDs correspond to absent or minimal enzyme activity, while attenuated and late-onset patients show residual enzyme activity, reflecting the underlying variants in the relevant gene. The residual enzyme activity may not be detected in leucocytes or dried blood spots but can often be measured in cultured skin fibroblasts. In some cases, particularly with adult patients, the enzyme activity may be just below the reference range and a highly suspicious clinical diagnosis may need further support from enzyme analysis in multiple sample types, as well as genetic and biomarker analysis. 

### 3.6. Biochemical Urine Tests

Biochemical urine tests for LSDs include GAGs, oligosaccharides, and sialic acid analyses, which should be considered when HSM is present. A full and complete investigation usually entails quantitative GAG analysis; GAG extraction; and two-dimensional low-voltage electrophoresis and thin layer chromatography (TLC) for oligosaccharides and sialic acids. As there are various qualitative (one- or two-dimensional electrophoresis) and quantitative methods available for the measurement of GAGs, all laboratories may not offer the same testing strategy and therefore clinicians should be aware of which disorders are being screened for at their referral laboratory of choice. For instance, some laboratories analyze quantitative GAGs and perform GAG extraction (Supplemental Figure S1) only when the initial test is positive. Therefore, clinicians should be aware of what is being offered at their referral center.

Increased amounts of quantitative GAGs in urine, as a screening method, may indicate MPS. However, it is known that attenuated MPS patients may not show this increase. Also, GAGs naturally decrease with age. Therefore, it is imperative for a laboratory to report against their own in-house validated age-related reference ranges. 

Excess oligosaccharides and sialic acid in urine can be detected using TLC. Again, there is variance across laboratories in which analyses are performed. For instance, the Willink laboratory performs TLC for oligosaccharides and sialic acids as standard on all urine samples arriving at the laboratory with a clinical suspicion of MPS. This strategy is used due to overlapping phenotypes for a variety of LSDs. Abnormal amounts of these compounds can give an indication of certain metabolic disorders such as Alpha mannosidosis, aspartylglucosaminuria, galactosialidosis, GM1 gangliosidosis, M\ucolipidosis II/III, neuraminidase deficiency and free sialic acid storage disease (Supplemental Figure S2). Similarly to GAGs, levels of intensity and detection of abnormal oligosaccharides and sialic acids decline with age. Results should always be confirmed by means of relevant enzymology and/or gene analysis of the appropriate indicated gene or genes.

In recent years, some laboratories have used liquid chromatography tandem mass spectrometry for the analysis of GAGs and oligosaccharides. These new methods, such as non-reducing end, appear to have a better performance with a higher sensitivity and specificity compared with those previously mentioned, although they are not widely available and are costly [91,92].

### 3.7. LSD Biomarkers

The LSD biomarkers currently available and emerging are listed in Table 2. Many novel biomarkers have been reported to have close to 100% sensitivity and specificity during initial evaluation, yet subsequently demonstrate limitations once used routinely. Studies reporting non-specific increases in LSD biomarkers are listed in Table 3; expanding collective understanding of these limitations is of high importance as the biomarker approach to laboratory diagnosis further proliferates.

The use of biomarkers in the LSD field is ever expanding and is frequently driven by the desire for high-throughput applications (e.g., newborn screening) or to overcome specific diagnostic challenges. To date, LSD biomarkers have found the highest value in situations where traditional enzymology cannot be used effectively (e.g., NPC, Cystinosis). Biomarkers can also be used to support more traditional enzymatic approaches to diagnosis of LSDs. For instance, equivocal leukocyte arylsulfatase A activity may be supported by the analysis of sulfatides; this may be of particular benefit to distinguish deficiencies (affected MLD) from pseudodeficiencies.

In some cases, analysis of a panel of biomarkers selected on the basis of the diagnostic performance and/or likely clinical overlap can improve specificity compared with analysis of an isolated biomarker. For example, N-palmitoyl-O-phosphocholineserine in combination with sphingosylphosphorylcholine and hexosyl-sphingosine were used for the differentiation of NPC, ASMD and Gaucher disease. Appropriate follow-up by means of enzymology can also be critical to achieving a definitive diagnosis via the exclusion of alternative causes of a biomarker pattern (e.g., measurement of lysosomal acid lipase activity in response to an increase in plasma oxysterol and/or N-palmitoyl-O-phosphocholineserine).

Biomarkers could prove to be helpful for the reclassification of variants of uncertain clinical significance, but this requires a holistic approach and careful consideration of all relevant biochemical, genetic and clinical factors.

### 3.8. Pathology

Pathology requires invasive procedures. Thus, biopsies should be avoided unless molecular testing or enzyme analysis are non-diagnostic or unavailable. Biopsies can be obtained from different tissues depending on the suspected LSDs. Skin biopsy is a minimally invasive source of fibroblasts which after being cultured can facilitate the diagnosis of certain LSDs based on enzyme levels or biomarkers. It can also be used to detect deposits in fibroblast and in other annex structures (glands, erector pili muscle, end terminal nerves). Patterns of filipin staining suggest NPC [134]. Sialic acid is elevated in the fibroblasts of patients with free sialic acid storage disease [135]. Biopsy of the subcutaneous nodules can show the typical “Farber bodies” [136].

Liver and muscle biopsies carry a higher risk of complications [57]. However, certain findings can help to narrow the differential, such as a positive PAS muscle biopsy, which is indicative of Pompe disease. Some findings in the liver biopsy are typical in lysosomal acid lipase deficiency like “sea-blue” histiocytes, vacuolated Kupffer cells, and cholesterol crystals [137]. ASMD presents with foamy cells and fibrosis [138]. “Gaucher cells” can be observed in liver, spleen or bone marrow biopsies [94].

Muscle biopsy may show secondary mitochondrial changes in LSDs [139]. Bone marrow biopsy is a test performed by hematologists when malignancy is suspected and is not thoroughly discussed in this review. 

## 4. Conclusions

HSM is a classical feature of several LSDs, and in some attenuated cases, it may be an isolated sign. Failure to consider LSDs in the setting of HSM impairs the rapid diagnosis and the commencement of therapy in a timely manner. While HSM is obvious in small children, adult patients may be identified incidentally with HSM by means of routine blood tests or imaging studies. It is worth considering a diagnosis of a LSD when all common hematological and general metabolic disorders have been excluded.

## Figures and Tables

**Table 1:** Lysosomal storage diseases associated with hepatomegaly and splenomegaly.

	Disease	Gene	Organomegaly
**Sphingolipidoses**	Gaucher disease [23]	*GBA*	++
	Acid Sphingomyelinase deficiency [24]	*SPMD1*	++
	Saposin C deficiency [25]	*PSAP*	++
	Farber disease (severe) [26] Farber disease (mild/intermediate) [26]	*ASAH1*	(+) (+/−)
	GM1 gangliosidosis type 1 [27] GM1 gangliosidosis type 2 [27]	*GLB1*	++ (+)
	Sandhoff disease (acute infantile) [28]	*HEXB*	(+/−)
	GM2 gangliosidosis, AB variant [29]	*GM2A*	(+/−)
	Multiple sulfatase deficiency [30]	*SUMF1*	+
**MPS**	Mucopolysaccharidosis I [31]	*IDUA*	++
	Mucopolysaccharidosis II [32]	*IDS* (X-linked)	+
	Mucopolysaccharidosis IIIA, IIIB [33]	*SGSH*, *NAGLU,*	+
	Mucopolysaccharidosis IIIC, IIID [34]	*HGSNAT*, *GNS*	(+/−)
	Mucopolysaccharidosis IVA, IVB [35]	*GLB1*, *GALNS*	(+)
	Mucopolysaccharidosis VI [36]	*ARSB*	(+)
	Mucopolysaccharidosis VII [37]	*GUSB*	(+)
**OLS**	Aspartylglucosaminuria [38]	*AGA*	(+/−)
	Fucosidosis [39]	*FUCA1*	(+)
	Galactosialidosis [40]	*CTSA*	++
	Alpha mannosidosis [41]	*MAN2B1*	+
	Schindler disease type III [42]	*NAGA*	(+/−)
	Neuraminidase deficiency type I [43] Neuraminidase deficiency type II [44]	*NEU1*	(+/−) +
**IMP**	Mucolipidosis II alpha/beta [45] Mucolipidosis III alpha/beta [45]	*GNPTAB*	++ (+)
	Nephropathic Cystinosis [46] Late-onset nephropathic cystinosis [47]	*CTNS*	+ (+/−)
	Free sialic acid storage disease [48]	*SLC17A5*	+
	Niemann-Pick type C [49]	*NPC1*, *NPC2*	(+)
**Other**	Lysosomal acid lipase deficiency (Wolman disease/CESD) [50]	*LIPA*	++
	Infantile onset Pompe disease [51]	*GAA*	+

++ Present usually, + present often, (+) present sometimes, (+/−) rarely present. CESD = cholesterol esterase storage disease; IMP = integral membrane proteins disorders; MPS = mucopolysaccharidoses; OLS = oligosaccharidosis.

**Table 2:** Diagnostic biomarkers of lysosomal storage diseases associated with hepatosplenomegaly.

Inherited Metabolic Disease	Specific Biomarker(s) *	Non-Specific Biomarkers **	Emerging Biomarkers
***Sphingolipidoses***			
Gaucher disease [93,94,95,96,97]	Glucosylsphingosine (lyso-GB1) ***	CCL18/PARC, glucosyl-cholesterol, Chito ****	
Combined saposin deficiency [98,99,100,101]	Glucosylsphingosine (lyso-GB1) ***, Galactosylsphingosine (psychosine), Globotriaosylsphingosine (lyso-GB3)	Chito ****	
Acid sphingomyelinase deficiency [102,103,104,105]	Plasma PPCS (↑↑–↑↑↑↑) with SPC (↑↑↑)	Plasma oxysterols (cholestane-3 β-5α-6β-triol, 7-ketocholesterol), Chito ****	
Farber disease [106]		Chito ****	C26:0 ceramide
GM1 gangliosidosis [99,107]	UO		Lyso-GM1, SUOL
Sandhoff disease [99,107,108]		Urinary DS trace amounts *****	Lyso-GM2, GM2 ganglioside, SUOL
Multiple sulfatase deficiency [30,109,110,111]		Urinary DS, HS, KS	Urinary/plasma sulfatides, SUOGL
***Mucopolysaccharidoses***			
MPS I [107,110,111]		Urinary DS, HS	SUOGL
MPS II [107,110,111]		Urinary DS, HS	SUOGL
MPS III [107,110,111]		Urinary HS	SUOGL
MPS IV [107,110,111]	Urinary KS		SUOGL
MPS VI [107,110,111]		Urinary DS	SUOGL
MPS VII [107,110,111]		Urinary DS, HS	SUOGL
***Oligosaccharidoses***			
Aspartylglucosaminuria [107]		Urinary aspartylglucosamine (ninhydrin detection), UO, urinary bound sialic acid	SUOL
Fucosidosis [107]			SUOL
Galactosialidosis [107]		US, UO	SUOL
Alpha mannosidosis [107]		UO	SUOL
Schindler disease type III [107]			SUOL
Neuraminidase deficiency [107]		US, UO	SUOL
***Integral membrane proteins disorders***			
Mucolipidosis II [107]	Lysosomal enzyme activities in plasma (↑↑)	Urinary DS trace amount *****, US *****	SUOL
Mucolipidosis III [107]	Lysosomal enzyme activities in plasma (↑↑)	Urinary DS trace amount *****, US *****	SUOL
Cystinosis [67]	Leukocyte cystine	Generalized amino aciduria (Fanconi syndrome)	
Infantile free sialic acid storage disease [107]		Urinary free sialic acid (N-acetylneuraminic acid)	SUOL
Niemann-Pick disease type C [93,98,99,102,103,104,112,113,114,115,116,117,118,119,120,121]	Plasma PPCS (↑–↑↑↑) with SPC (N-↑)	Plasma oxysterols (cholestane-3β, 5α, 6β-triol, 7-ketocholesterol), Glucosyl-cholesterol, Chito ****	N-(3β,5α,6β-trihydroxy-cholan-24-oyl) glycine Urinary sulphate—conjugated cholesterol metabolites (bile acids)
***Others***			
Lysosomal acid lipase deficiency [112,113]		↑↑↑ Plasma oxysterols (cholestane-3β,5α,6β-triol, 7-ketocholesterol), Chito ****	
Pompe disease [122]		UO (tetra) *****	Urinary glucose tetrasaccharide (Glc4)

Chito = chitotriosidase; DS = dermatan sulphate; HS = heparan sulphate; KS = keratan sulphate; LC-MS/MS = liquid chromatography tandem mass spectrometry; MPS = mucopolysaccharidosis; PPCS = N-palmitoyl-O-phosphocholineserine (formerly lyso-sphingomyelin-509); SPC = sphingosylphosphorylcholine (otherwise known as lyso-sphingomyelin); SUOGL = specific urinary oligosaccharide GAG fragments detected by LC-MS/MS; SUOL = specific urinary oligosaccharides detected by LC-MS/MS; UO = urinary oligosaccharide; US = urinary bound sialic acid. * For disorders caused by enzyme deficiencies, sensitive and specific laboratory diagnosis can be achieved by measuring the activity of the affected enzyme. Biomarker specificity is often <100%; see Table 3 for other disease states/factors known to yield increases. ** Note: thin layer chromatography for oligosaccharides and sialic acids is not as sensitive or specific as the more recently introduced tandem mass spectrometry methods for biomarkers which may be stored in these disorders. Certain disorders detected with biomarkers in newly developed assays will not necessarily be detected by thin layer chromatography. Specific knowledge of local laboratory methods is required. *** Often measured as total hexosyl-sphingosine (glucosyl + galactosyl). **** Ineffective clinical sensitivity in 4–6% of general population due to chitotriosidase deficiency. Increases in chitotriosidase are typically exaggerated in Gaucher disease (↑↑–↑↑↑↑) but variable dependent on wild type/heterozygous CHIT1 deficiency status [18]. In other LSDs, the clinical sensitivity of chitotriosidase activity is sub-optimal. Chitotriosidase activity is a non-specific marker of macrophage activation and thus not a suitable first line stand-alone screen test for LSDs. ***** Dependent on the methodology used, the biomarkers detected may vary due to the sensitivity of the method. For example, traces of dermatan sulphate may be detected in some patients with mucolipidosis II/III and Sandhoff, but not all. One arrow means that it is slightly raised, 2-3 arrows reflect the moderate increase, four arrows reflects the significant rise.

**Table 3:** Literature-reported non-specific increases in LSD biomarkers.

Biomarker	Primary Diagnostic Utility	Non-Specific Increases
Cholestane-3β,5α,6β-triol (C-triol) [112,113,120,121,123,124]	NPC	NPC heterozygotes, ASMD, LALD, INCL, Gaucher disease, CTX, cholestasis, hypercholesterolemia, sub-optimal sample handling (ex-vivo auto-oxidation of cholesterol)
7-ketocholesterol [113,120,121,125]	NPC	NPC heterozygotes, ASMD, LALD, INCL, Gaucher disease, CTX, cholestasis, hypercholesterolemia, sub-optimal sample handling (ex-vivo auto-oxidation of cholesterol)
PPCS (lyso-sphingomyelin-509) [98,99,102,103,115,116,117,126]	NPC *	ASMD, LALD, Gaucher disease, CDGs
Lyso-sphingomyelin (SPC) [98,99,126,127]	ASMD *	NPC, Gaucher disease, peroxisomal disorders
Hexosyl-sphingosine (glucosyl + galactosyl) [94,95,98,99,126,127]	Gaucher disease *	Krabbe disease, NPC, Fabry disease
Glucosylsphingosine (Lyso-GB1) [94,95,98,99,126,127]	Gaucher disease *	NPC, Fabry disease
Urinary glucose tetrasaccharide [122,128,129,130]	Pompe disease	GSDIII, GSDVI, GSDIX, DMD, muscle trauma, pregnancy, cancers.
Urinary aspartylglucosamine (ASG) [131,132]	Aspartylglucosaminuria	NGLY1 deficiency
Chitotriosidase activity **	Supportive of gold standard enzyme assays	Many
Urinary free sialic acid [133]	Free sialic acid storage disease, sialuria	Diabetes mellitus, HUS, renal dysfunction
Urinary bound sialic acid [133]	Galactosialidosis, sialidosis	Diabetes mellitus, HUS, renal dysfunction

ASMD = acid sphingomyelinase deficiency; CDGs = congenital disorders of glycosylation; CTX = cerebrotendinous xanthomatosis; DMD = Duchene muscular dystrophy; GSD = glycogen storage disease; HUS = hemolytic uremic syndrome; INCL = infantile neuronal ceroid lipofuscinosis; LALD = lysosomal acid lipase deficiency; NPC = Niemann–Pick disease type C; PPCS = N-palmitoyl-O-phosphocholineserine. * Analysis of multiple lyso-sphingolipid species as a panel improved clinical specificity, e.g., ASMD and NPC may be differentiated (where PPCS is increased) based on the degree of increase in lyso-sphingomyelin. Gaucher disease can be differentiated from NPC by the demonstration of a gross increase in hexosyl-sphingosine. Krabbe disease can be differentiated from Gaucher disease by the chromatographic resolution of glucosyl and galactosyl (psychosine) isomers. ** Chitotriosidase activity is a general, non-specific marker of inflammation and macrophage activation with increases demonstrated in many LSDs along with a wide variety of other pathologies; a comprehensive list is considered beyond the scope of this review.

## Supplementary Materials

The following supporting information can be downloaded at: https://www.mdpi.com/article/10.3390/jcm13051465/s1, Figure S1: Two-dimensional electrophoresis urine GAGs. A. Normal, only CS present. B. Attenuated Mucopolysaccharidosis type VI, less DS than CS. C. Mucopolysaccharidosis type VI, more DS than CS; Figure S2: Oligosaccharide Thin Layer Chromatography Plate.

## Abbreviations

ASMD	Acid sphingomyelinase deficiency
CESD	Cholesterol esterase storage disease
ERT	Enzyme replacement therapy
GAGs	Glycosaminoglycans
HSCT	Haematopoietic stem cell transplantation
HSM	Hepatosplenomegaly
IMDs	Inherited metabolic disorders
LSDs	Lysosomal storage diseases
NPC	Niemann–Pick disease type C
MPS	Mucopolysaccharidosis
TLC	Thin layer chromatography

## References

[1] Loloi J., Patel A., McDevitt P., Bruno M.A., Riley T. (2019). How Strongly Do Physical Examination Estimates and Ultrasonographic Measurements of Liver Size Correlate? A Prospective Study. Am. J. Med..

[2] Olson A.P.J., Trappey B., Wagner M., Newman M., Nixon L.J., Schnobrich D. (2015). Point-of-Care Ultrasonography Improves the Diagnosis of Splenomegaly in Hospitalized Patients. Crit. Ultrasound J..

[3] Joshi R., Singh A., Jajoo N., Pai M., Kalantri S.P. (2004). Accuracy and Reliability of Palpation and Percussion for Detecting Hepatomegaly: A Rural Hospital-Based Study. Indian J. Gastroenterol..

[4] Childs J.T., Esterman A.J., Thoirs K.A., Turner R.C. (2016). Ultrasound in the Assessment of Hepatomegaly: A Simple Technique to Determine an Enlarged Liver Using Reliable and Valid Measurements. Sonography.

[5] Curovic Rotbain E., Lund Hansen D., Schaffalitzky de Muckadell O., Wibrand F., Meldgaard Lund A., Frederiksen H. (2017). Splenomegaly—Diagnostic Validity, Work-up, and Underlying Causes. PLoS ONE.

[6] Benzamin M., Sayeed M., Karim A.S.M., Rukunuzzaman M. (2018). Study of Etiological Profile of Children Presented with Hepatomegaly and/or Splenomegaly: An Experience from Pediatric Gastroenterology Department, Bangabandhu Sheikh Mujib Medical University. Paediatr. Nephrol. J. Bangladesh.

[7] Ferreira C.R., Cassiman D., Blau N. (2019). Clinical and Biochemical Footprints of Inherited Metabolic Diseases. II. Metabolic Liver Diseases. Mol. Genet Metab.

[8] Baker A. (2017). Hepatosplenomegaly. Paediatr. Child Health.

[9] Kavanagh K., Pastores G.M. (2021). Hepatic Manifestations of Lysosomal Storage Disorders: Differential Diagnosis, Investigations, and Treatment, Current and Upcoming. EMJ.

[10] Platt F.M., d’Azzo A., Davidson B.L., Neufeld E.F., Tifft C.J. (2018). Lysosomal Storage Diseases. Nat. Rev. Dis. Primers.

[11] Li P., Gu M., Xu H. (2019). Lysosomal Ion Channels as Decoders of Cellular Signals. Trends Biochem. Sci..

[12] Stepien K.M., Kieć-Wilk B., Lampe C., Tangeraas T., Cefalo G., Belmatoug N., Francisco R., del Toro M., Wagner L., Lauridsen A.-G. (2021). Challenges in Transition From Childhood to Adulthood Care in Rare Metabolic Diseases: Results From the First Multi-Center European Survey. Front. Med..

[13] Fernández-Pereira C., San Millán-Tejado B., Gallardo-Gómez M., Pérez-Márquez T., Alves-Villar M., Melcón-Crespo C., Fernández-Martín J., Ortolano S. (2021). Therapeutic Approaches in Lysosomal Storage Diseases. Biomolecules.

[14] Lachmann R.H., Diaz G.A., Wasserstein M.P., Armstrong N.M., Yarramaneni A., Kim Y., Kumar M. (2023). Olipudase Alfa Enzyme Replacement Therapy for Acid Sphingomyelinase Deficiency (ASMD): Sustained Improvements in Clinical Outcomes after 6.5 Years of Treatment in Adults. Orphanet J. Rare Dis..

[15] Mistry P.K., Lukina E., Ben Turkia H., Shankar S.P., Baris Feldman H., Ghosn M., Mehta A., Packman S., Lau H., Petakov M. (2021). Clinical Outcomes after 4.5 Years of Eliglustat Therapy for Gaucher Disease Type 1: Phase 3 ENGAGE Trial Final Results. Am. J. Hematol..

[16] Ayto R.M., Hughes D.A., Jeevaratnam P., Rolles K., Burroughs A.K., Mistry P.K., Mehta A.B., Pastores G.M. (2010). Long-Term Outcomes of Liver Transplantation in Type 1 Gaucher Disease. Am. J. Transpl..

[17] Patterson M.C., Vecchio D., Prady H., Abel L., Wraith J.E. (2007). Miglustat for Treatment of Niemann-Pick C Disease: A Randomised Controlled Study. Lancet Neurol..

[18] Naumchik B.M., Gupta A., Flanagan-Steet H., Steet R.A., Cathey S.S., Orchard P.J., Lund T.C. (2020). The Role of Hematopoietic Cell Transplant in the Glycoprotein Diseases. Cells.

[19] Hoffmann G.F., McKiernan P., Hoffmann G.F., Zschocke J., Nyhan W.L. (2017). Liver Disease. Inherited Metabolic Diseases: A Clinical Approach.

[20] Thipphavong S., Duigenan S., Schindera S.T., Gee M.S., Philips S. (2014). Nonneoplastic, Benign, and Malignant Splenic Diseases: Cross-Sectional Imaging Findings and Rare Disease Entities. Am. J. Roentgenol..

[21] vom Dahl S., Mengel E. (2010). Lysosomal Storage Diseases as Differential Diagnosis of Hepatosplenomegaly. Best Pract. Res. Clin. Gastroenterol..

[22] La Cognata V., Guarnaccia M., Polizzi A., Ruggieri M., Cavallaro S. (2020). Highlights on Genomics Applications for Lysosomal Storage Diseases. Cells.

[23] Stirnemann J., Belmatoug N., Camou F., Serratrice C., Froissart R., Caillaud C., Levade T., Astudillo L., Serratrice J., Brassier A. (2017). A Review of Gaucher Disease Pathophysiology, Clinical Presentation and Treatments. Int. J. Mol. Sci..

[24] McGovern M.M., Wasserstein M.P., Bembi B., Giugliani R., Mengel K.E., Vanier M.T., Zhang Q., Peterschmitt M.J. (2021). Prospective Study of the Natural History of Chronic Acid Sphingomyelinase Deficiency in Children and Adults: Eleven Years of Observation. Orphanet J. Rare Dis..

[25] Liaqat K., Hussain S., Acharya A., Nasir A., Bharadwaj T., Ansar M., Basit S., Schrauwen I., Ahmad W., Leal S.M. (2022). Phenotype Expansion for Atypical Gaucher Disease Due to Homozygous Missense PSAP Variant in a Large Consanguineous Pakistani Family. Genes.

[26] Yu F.P.S., Amintas S., Levade T., Medin J.A. (2018). Acid Ceramidase Deficiency: Farber Disease and SMA-PME. Orphanet J. Rare Dis..

[27] Lang F.M., Korner P., Harnett M., Karunakara A., Tifft C.J. (2020). The Natural History of Type 1 Infantile GM1 Gangliosidosis: A Literature-Based Meta-Analysis. Mol. Genet. Metab..

[28] Karimzadeh P., Jafari N., Nejad Biglari H., Jabbeh Dari S., Ahmad Abadi F., Alaee M.-R., Nemati H., Saket S., Tonekaboni S.H., Taghdiri M.-M. (2014). GM2-Gangliosidosis (Sandhoff and Tay Sachs Disease): Diagnosis and Neuroimaging Findings (An Iranian Pediatric Case Series). Iran J. Child Neurol..

[29] İnci A., Ergin F.B.C., Biberoğlu G., Okur İ., Ezgü F.S., Tümer L. (2021). Two Patients from Turkey with a Novel Variant in the GM2A Gene and Review of the Literature. J. Pediatr. Endocrinol. Metab..

[30] Schlotawa L., Adang L.A., Radhakrishnan K., Ahrens-Nicklas R.C. (2020). Multiple Sulfatase Deficiency: A Disease Comprising Mucopolysaccharidosis, Sphingolipidosis, and More Caused by a Defect in Posttranslational Modification. Int. J. Mol. Sci..

[31] Clarke L.A., Adam M.P., Mirzaa G.M., Pagon R.A., Wallace S.E., Bean L.J., Gripp K.W., Amemiya A. (1993). Mucopolysaccharidosis Type I. GeneReviews^®^.

[32] Hampe C.S., Yund B.D., Orchard P.J., Lund T.C., Wesley J., McIvor R.S. (2021). Differences in MPS I and MPS II Disease Manifestations. Int. J. Mol. Sci..

[33] Zelei T., Csetneki K., Vokó Z., Siffel C. (2018). Epidemiology of Sanfilippo Syndrome: Results of a Systematic Literature Review. Orphanet J. Rare Dis..

[34] Jones M.Z., Alroy J., Rutledge J.C., Taylor J.W., Alvord E.C., Toone J., Applegarth D., Hopwood J.J., Skutelsky E., Ianelli C. (1997). Human Mucopolysaccharidosis IIID: Clinical, Biochemical, Morphological and Immunohistochemical Characteristics. J. Neuropathol. Exp. Neurol..

[35] Hendriksz C.J., Berger K.I., Giugliani R., Harmatz P., Kampmann C., Mackenzie W.G., Raiman J., Villarreal M.S., Savarirayan R. (2015). International Guidelines for the Management and Treatment of Morquio A Syndrome. Am. J. Med. Genet. Part A.

[36] Kılıç M., Dursun A., Coşkun T., Tokatlı A., Özgül R.K., Yücel-Yılmaz D., Karaca M., Doğru D., Alehan D., Kadayıfçılar S. (2017). Genotypic-Phenotypic Features and Enzyme Replacement Therapy Outcome in Patients with Mucopolysaccharidosis VI from Turkey. Am. J. Med. Genet. Part A.

[37] Zielonka M., Garbade S.F., Kölker S., Hoffmann G.F., Ries M. (2017). Quantitative Clinical Characteristics of 53 Patients with MPS VII: A Cross-Sectional Analysis. Genet. Med..

[38] Selvanathan A., Kinsella J., Moore F., Wynn R., Jones S., Shaw P.J., Wilcken B., Bhattacharya K. (2021). Effectiveness of Early Hematopoietic Stem Cell Transplantation in Preventing Neurocognitive Decline in Aspartylglucosaminuria: A Case Series. JIMD Rep..

[39] Stepien K.M., Ciara E., Jezela-Stanek A. (2020). Fucosidosis—Clinical Manifestation, Long-Term Outcomes, and Genetic Profile—Review and Case Series. Genes.

[40] Alsahlawi Z., Aljishi E., Kheyami A., Alekri A., Alwedaie S.M.J. (2022). Clinical Spectrum and Outcome of Nine Patients with a Novel Genetic Variant of Galactosialidosis in the Kingdom of Bahrain. JIMD Rep..

[41] Lipiński P., Różdżyńska-Świątkowska A., Iwanicka-Pronicka K., Perkowska B., Pokora P., Tylki-Szymańska A. (2022). Long-Term Outcome of Patients with Alpha-Mannosidosis—A Single Center Study. Mol. Genet. Metab. Rep..

[42] Chabás A., Duque J., Gort L. (2007). A New Infantile Case of α-N-Acetylgalactosaminidase Deficiency. Cardiomyopathy as a Presenting Symptom. J. Inherit. Metab. Dis..

[43] Caciotti A., Melani F., Tonin R., Cellai L., Catarzi S., Procopio E., Chilleri C., Mavridou I., Michelakakis H., Fioravanti A. (2020). Type I Sialidosis, a Normosomatic Lysosomal Disease, in the Differential Diagnosis of Late-Onset Ataxia and Myoclonus: An Overview. Mol. Genet. Metab..

[44] Caciotti A., Di Rocco M., Filocamo M., Grossi S., Traverso F., d’Azzo A., Cavicchi C., Messeri A., Guerrini R., Zammarchi E. (2009). Type II Sialidosis: Review of the Clinical Spectrum and Identification of a New Splicing Defect with Chitotriosidase Assessment in Two Patients. J. Neurol..

[45] Otomo T., Muramatsu T., Yorifuji T., Okuyama T., Nakabayashi H., Fukao T., Ohura T., Yoshino M., Tanaka A., Okamoto N. (2009). Mucolipidosis II and III Alpha/Beta: Mutation Analysis of 40 Japanese Patients Showed Genotype–Phenotype Correlation. J. Hum. Genet..

[46] Topaloglu R., Gültekingil A., Gülhan B., Ozaltin F., Demir H., Çiftci T., Demir N., Temucin Ç.M., Yuce A., Akhan O. (2020). Cystinosis beyond Kidneys: Gastrointestinal System and Muscle Involvement. BMC Gastroenterol..

[47] Nakhaie S., Sharif A.S., Hosseini Shamsabadi R., Otukesh H., Hashemipour M., Mohammadi S. (2022). Gastrointestinal Manifestations of Adult Cystinosis in Iran: A Descriptive Study. Med. J. Islam Repub. Iran.

[48] Adams D., Wasserstein M., Adam M.P., Mirzaa G.M., Pagon R.A., Wallace S.E., Bean L.J., Gripp K.W., Amemiya A. (1993). Free Sialic Acid Storage Disorders. GeneReviews^®^.

[49] Patterson M.C., Mengel E., Vanier M.T., Moneuse P., Rosenberg D., Pineda M. (2020). Treatment Outcomes Following Continuous Miglustat Therapy in Patients with Niemann-Pick Disease Type C: A Final Report of the NPC Registry. Orphanet J. Rare Dis..

[50] Burton B.K., Deegan P.B., Enns G.M., Guardamagna O., Horslen S., Hovingh G.K., Lobritto S.J., Malinova V., McLin V.A., Raiman J. (2015). Clinical Features of Lysosomal Acid Lipase Deficiency. J. Pediatr. Gastroenterol. Nutr..

[51] Leslie N., Bailey L., Adam M.P., Mirzaa G.M., Pagon R.A., Wallace S.E., Bean L.J., Gripp K.W., Amemiya A. (1993). Pompe Disease. GeneReviews^®^.

[52] Bulut F.D., BìLgìNer Gürbüz B. (2022). Etiological Evaluation of Patients with Hepatomegaly, Splenomegaly and Hepatosplenomegaly Referred to a Pediatric Metabolism Unit. Acibadem. Univ. Saglik Bilim Derg..

[53] Jensen K.K., Oh K.Y., Patel N., Narasimhan E.R., Ku A.S., Sohaey R. (2020). Fetal Hepatomegaly: Causes and Associations. RadioGraphics.

[54] Xiao C., Koziura M., Cope H., Spillman R., Tan K., Hisama F.M., Tifft C.J., Toro C. (2022). Adults with Lysosomal Storage Diseases in the Undiagnosed Diseases Network. Mol. Genet. Genom. Med..

[55] Hazan G., Hershkovitz E., Staretz-Chacham O. (2020). Incidence of Inherited Metabolic Disorders in Southern Israel: A Comparison between Consanguinity and Non-Consanguinity Communities. Orphanet J. Rare Dis..

[56] McBride K.L., Flanigan K.M. (2021). Update in the Mucopolysaccharidoses. Semin. Pediatr. Neurol..

[57] Ferreira C.R., Gahl W.A. (2017). Lysosomal Storage Diseases. Transl. Sci. Rare Dis..

[58] Senarathne U.D., Indika N.-L.R., Jezela-Stanek A., Ciara E., Frye R.E., Chen C., Stepien K.M. (2023). Biochemical, Genetic and Clinical Diagnostic Approaches to Autism-Associated Inherited Metabolic Disorders. Genes.

[59] Sedel F., Baumann N., Turpin J.-C., Lyon-Caen O., Saudubray J.-M., Cohen D. (2007). Psychiatric Manifestations Revealing Inborn Errors of Metabolism in Adolescents and Adults. J. Inherit. Metab. Dis..

[60] Oelsner K., Adams M.E., Sarwark J.F., Carl R.L. (2023). Musculoskeletal Manifestations of Mucopolysaccharidoses. Orthopaedics for the Newborn and Young Child: A Practical Clinical Guide.

[61] Nestrasil I., Ahmed A., Utz J.M., Rudser K., Whitley C.B., Jarnes-Utz J.R. (2018). Distinct Progression Patterns of Brain Disease in Infantile and Juvenile Gangliosidoses: Volumetric Quantitative MRI Study. Mol. Genet. Metab..

[62] Ferreira C.R., Martinelli D., Blau N. (2021). Clinical and Biochemical Footprints of Inherited Metabolic Diseases. VI. Metabolic Dermatoses. Mol. Genet. Metab..

[63] Hanson M., Lupski J.R., Hicks J., Metry D. (2003). Association of Dermal Melanocytosis With Lysosomal Storage Disease: Clinical Features and Hypotheses Regarding Pathogenesis. Arch. Dermatol..

[64] Stepien K.M., Bentley A., Chen C., Dhemech M.W., Gee E., Orton P., Pringle C., Rajan J., Saxena A., Tol G. (2022). Non-Cardiac Manifestations in Adult Patients With Mucopolysaccharidosis. Front. Cardiovasc. Med..

[65] Maroofian R., Schuele I., Najafi M., Bakey Z., Rad A., Antony D., Habibi H., Schmidts M. (2018). Parental Whole-Exome Sequencing Enables Sialidosis Type II Diagnosis Due to an NEU1 Missense Mutation as an Underlying Cause of Nephrotic Syndrome in the Child. Kidney Int. Rep..

[66] Steinke J., Gessner M., Frauenfeld L., Fischer A.K., Solass W. (2020). Loss of Kidney Function Due to Proteinuria, Common Problem with a Rare Cause: Answer. Pediatr. Nephrol..

[67] Elmonem M.A., Veys K.R., Soliman N.A., van Dyck M., van den Heuvel L.P., Levtchenko E. (2016). Cystinosis: A Review. Orphanet J. Rare Dis..

[68] Tripathy K., Patel B.C. (2023). Cherry Red Spot. StatPearls.

[69] Fenzl C.R., Teramoto K., Moshirfar M. (2015). Ocular Manifestations and Management Recommendations of Lysosomal Storage Disorders I: Mucopolysaccharidoses. Clin. Ophthalmol..

[70] Salsano E., Umeh C., Rufa A., Pareyson D., Zee D.S. (2012). Vertical Supranuclear Gaze Palsy in Niemann-Pick Type C Disease. Neurol. Sci..

[71] Eghbali A., Hassan S., Seehra G., FitzGibbon E., Sidransky E. (2019). Ophthalmological Findings in Gaucher Disease. Mol. Genet. Metab..

[72] Chis B.A., Ismaiel A., Chis A.-F. (2023). Hepatic, Splenic, and Bone Marrow Gaucheromas: A Case Series and Systematic Literature Review. J. Gastrointestin Liver Dis..

[73] Marshall W.C., Ockenden B.G., Fosbrooke A.S., Cumings J.N. (1969). Wolman’s Disease. A Rare Lipidosis with Adrenal Calcification. Arch. Dis. Child.

[74] Nascimbeni F., Lugari S., Cassinerio E., Motta I., Cavicchioli A., Dalla Salda A., Bursi S., Donatiello S., Spina V., Cappellini M.D. (2020). Liver Steatosis Is Highly Prevalent and Is Associated with Metabolic Risk Factors and Liver Fibrosis in Adult Patients with Type 1 Gaucher Disease. Liver Int..

[75] Lipiński P., Szymańska-Rożek P., Socha P., Tylki-Szymańska A. (2020). Controlled Attenuation Parameter and Liver Stiffness Measurements Using Transient Elastography by FibroScan in Gaucher Disease. Mol. Genet. Metab..

[76] Hughes D., Mikosch P., Belmatoug N., Carubbi F., Cox T., Goker-Alpan O., Kindmark A., Mistry P., Poll L., Weinreb N. (2019). Gaucher Disease in Bone: From Pathophysiology to Practice. J. Bone Min. Res..

[77] Wasserstein M., Godbold J., McGovern M.M. (2013). Skeletal Manifestations in Pediatric and Adult Patients with Niemann Pick Disease Type B. J. Inherit. Metab. Dis..

[78] Dardis A., Michelakakis H., Rozenfeld P., Fumic K., Wagner J., Pavan E., Fuller M., Revel-Vilk S., Hughes D., Cox T. (2022). Patient Centered Guidelines for the Laboratory Diagnosis of Gaucher Disease Type 1. Orphanet J. Rare Dis..

[79] Reiner Ž., Guardamagna O., Nair D., Soran H., Hovingh K., Bertolini S., Jones S., Ćorić M., Calandra S., Hamilton J. (2014). Lysosomal Acid Lipase Deficiency—An under-Recognized Cause of Dyslipidaemia and Liver Dysfunction. Atherosclerosis.

[80] Anderson G., Smith V.V., Malone M., Sebire N.J. (2005). Blood Film Examination for Vacuolated Lymphocytes in the Diagnosis of Metabolic Disorders; Retrospective Experience of More than 2500 Cases from a Single Centre. J. Clin. Pathol..

[81] Nava-Aguilera M.-L., Velasco-Rodríguez D., Villarrubia J., Alonso-Domínguez J.-M., Carrillo-Farga J. (2014). Azurophilic Inclusions in Lymphocytes and Plasma Cells: A Case of Sanfilippo Disease. Br. J. Haematol..

[82] Zanetti A., D’Avanzo F., Bertoldi L., Zampieri G., Feltrin E., De Pascale F., Rampazzo A., Forzan M., Valle G., Tomanin R. (2020). Setup and Validation of a Targeted Next-Generation Sequencing Approach for the Diagnosis of Lysosomal Storage Disorders. J. Mol. Diagn..

[83] Bean L.J.H., Funke B., Carlston C.M., Gannon J.L., Kantarci S., Krock B.L., Zhang S., Bayrak-Toydemir P. (2020). Diagnostic Gene Sequencing Panels: From Design to Report—A Technical Standard of the American College of Medical Genetics and Genomics (ACMG). Genet. Med..

[84] McDermott H., Sherlaw-Sturrock C., Baptista J., Hartles-Spencer L., Naik S. (2022). Rapid Exome Sequencing in Critically Ill Children Impacts Acute and Long-Term Management of Patients and Their Families: A Retrospective Regional Evaluation. Eur. J. Med. Genet..

[85] Lincoln S.E., Hambuch T., Zook J.M., Bristow S.L., Hatchell K., Truty R., Kennemer M., Shirts B.H., Fellowes A., Chowdhury S. (2021). One in Seven Pathogenic Variants Can Be Challenging to Detect by NGS: An Analysis of 450,000 Patients with Implications for Clinical Sensitivity and Genetic Test Implementation. Genet. Med..

[86] Strovel E.T., Cusmano-Ozog K., Wood T., Yu C., ACMG Laboratory Quality Assurance Committee (2022). Measurement of Lysosomal Enzyme Activities: A Technical Standard of the American College of Medical Genetics and Genomics (ACMG). Genet. Med..

[87] Sláma T., Garbade S.F., Kölker S., Hoffmann G.F., Ries M. (2019). Quantitative Natural History Characterization in a Cohort of 142 Published Cases of Patients with Galactosialidosis—A Cross-Sectional Study. J. Inherit. Metab. Dis..

[88] Vanier M.T., Latour P. (2015). Laboratory Diagnosis of Niemann-Pick Disease Type C: The Filipin Staining Test. Methods Cell Biol..

[89] Adang L.A., Schlotawa L., Groeschel S., Kehrer C., Harzer K., Staretz-Chacham O., Silva T.O., Schwartz I.V.D., Gärtner J., De Castro M. (2020). Natural History of Multiple Sulfatase Deficiency: Retrospective Phenotyping and Functional Variant Analysis to Characterize an Ultra-Rare Disease. J. Inherit. Metab. Dis..

[90] van Diggelen O.P., Voznyi Y.V., Keulemans J.L.M., Schoonderwoerd K., Ledvinova J., Mengel E., Zschiesche M., Santer R., Harzer K. (2005). A New Fluorimetric Enzyme Assay for the Diagnosis of Niemann-Pick A/B, with Specificity of Natural Sphingomyelinase Substrate. J. Inherit. Metab. Dis..

[91] Al Dhahouri N., Langhans C.-D., Al Hammadi Z., Okun J.G., Hoffmann G.F., Al-Jasmi F., Al-Dirbashi O.Y. (2018). Quantification of Methylcitrate in Dried Urine Spots by Liquid Chromatography Tandem Mass Spectrometry for the Diagnosis of Propionic and Methylmalonic Acidemias. Clin. Chim. Acta.

[92] Vera M.U., Le S.Q., Victoroff A., Passage M.B., Brown J.R., Crawford B.E., Polgreen L.E., Chen A.H., Dickson P.I. (2020). Evaluation of Non-Reducing End Pathologic Glycosaminoglycan Detection Method for Monitoring Therapeutic Response to Enzyme Replacement Therapy in Human Mucopolysaccharidosis I. Mol. Genet. Metab..

[93] Marques A.R.A., Mirzaian M., Akiyama H., Wisse P., Ferraz M.J., Gaspar P., Ghauharali-van der Vlugt K., Meijer R., Giraldo P., Alfonso P. (2016). Glucosylated Cholesterol in Mammalian Cells and Tissues: Formation and Degradation by Multiple Cellular β-Glucosidases. J. Lipid Res..

[94] Dekker N., van Dussen L., Hollak C.E.M., Overkleeft H., Scheij S., Ghauharali K., van Breemen M.J., Ferraz M.J., Groener J.E.M., Maas M. (2011). Elevated Plasma Glucosylsphingosine in Gaucher Disease: Relation to Phenotype, Storage Cell Markers, and Therapeutic Response. Blood.

[95] Giuffrida G., Markovic U., Condorelli A., Calafiore V., Nicolosi D., Calagna M., Grasso S., Ragusa M.T.V., Gentile J., Napolitano M. (2023). Glucosylsphingosine (Lyso-Gb1) as a Reliable Biomarker in Gaucher Disease: A Narrative Review. Orphanet J. Rare Dis..

[96] Hollak C.E., van Weely S., van Oers M.H., Aerts J.M. (1994). Marked Elevation of Plasma Chitotriosidase Activity. A Novel Hallmark of Gaucher Disease. J. Clin. Invest.

[97] Boot R.G., Verhoek M., de Fost M., Hollak C.E.M., Maas M., Bleijlevens B., van Breemen M.J., van Meurs M., Boven L.A., Laman J.D. (2004). Marked Elevation of the Chemokine CCL18/PARC in Gaucher Disease: A Novel Surrogate Marker for Assessing Therapeutic Intervention. Blood.

[98] Polo G., Burlina A.P., Kolamunnage T.B., Zampieri M., Dionisi-Vici C., Strisciuglio P., Zaninotto M., Plebani M., Burlina A.B. (2017). Diagnosis of Sphingolipidoses: A New Simultaneous Measurement of Lysosphingolipids by LC-MS/MS. Clin. Chem. Lab. Med..

[99] Pettazzoni M., Froissart R., Pagan C., Vanier M.T., Ruet S., Latour P., Guffon N., Fouilhoux A., Germain D.P., Levade T. (2017). LC-MS/MS Multiplex Analysis of Lysosphingolipids in Plasma and Amniotic Fluid: A Novel Tool for the Screening of Sphingolipidoses and Niemann-Pick Type C Disease. PLoS ONE.

[100] Motta M., Tatti M., Furlan F., Celato A., Di Fruscio G., Polo G., Manara R., Nigro V., Tartaglia M., Burlina A. (2016). Clinical, Biochemical and Molecular Characterization of Prosaposin Deficiency. Clin. Genet..

[101] Harzer K., Hiraiwa M., Paton B.C. (2001). Saposins (Sap) A and C Activate the Degradation of Galactosylsphingosine. FEBS Lett..

[102] Sidhu R., Kell P., Dietzen D.J., Farhat N.Y., Do A.N.D., Porter F.D., Berry-Kravis E., Vite C.H., Reunert J., Marquardt T. (2020). Application of N-Palmitoyl-O-Phosphocholineserine for Diagnosis and Assessment of Response to Treatment in Niemann-Pick Type C Disease. Mol. Genet. Metab..

[103] Voorink-Moret M., Goorden S.M.I., van Kuilenburg A.B.P., Wijburg F.A., Ghauharali-van der Vlugt J.M.M., Beers-Stet F.S., Zoetekouw A., Kulik W., Hollak C.E.M., Vaz F.M. (2018). Rapid Screening for Lipid Storage Disorders Using Biochemical Markers. Expert Center Data and Review of the Literature. Mol. Genet. Metab..

[104] Iwahori A., Maekawa M., Narita A., Kato A., Sato T., Ogura J., Sato Y., Kikuchi M., Noguchi A., Higaki K. (2020). Development of a Diagnostic Screening Strategy for Niemann-Pick Diseases Based on Simultaneous Liquid Chromatography-Tandem Mass Spectrometry Analyses of N-Palmitoyl-O-Phosphocholine-Serine and Sphingosylphosphorylcholine. Biol. Pharm. Bull..

[105] Kubaski F., Burlina A., Pereira D., Silva C., Herbst Z.M., Trapp F.B., Michelin-Tirelli K., Lopes F.F., Burin M.G., Brusius-Facchin A.C. (2022). Quantification of Lysosphingomyelin and Lysosphingomyelin-509 for the Screening of Acid Sphingomyelinase Deficiency. Orphanet J. Rare Dis..

[106] Cozma C., Iurașcu M.-I., Eichler S., Hovakimyan M., Brandau O., Zielke S., Böttcher T., Giese A.-K., Lukas J., Rolfs A. (2017). C26-Ceramide as Highly Sensitive Biomarker for the Diagnosis of Farber Disease. Sci. Rep..

[107] Mak J., Cowan T.M. (2021). Detecting Lysosomal Storage Disorders by Glycomic Profiling Using Liquid Chromatography Mass Spectrometry. Mol. Genet. Metab..

[108] Blondel A., Kraoua I., Marcelino C., Khrouf W., Schlemmer D., Ganne B., Caillaud C., Fernández-Eulate G., Turki I.B.Y., Dauriat B. (2023). Plasma GM2 Ganglioside Potential Biomarker for Diagnosis, Prognosis and Disease Monitoring of GM2-Gangliosidosis. Mol. Genet. Metab..

[109] Beck-Wödl S., Kehrer C., Harzer K., Haack T.B., Bürger F., Haas D., Rieß A., Groeschel S., Krägeloh-Mann I., Böhringer J. (2021). Long-Term Disease Course of Two Patients with Multiple Sulfatase Deficiency Differs from Metachromatic Leukodystrophy in a Broad Cohort. JIMD Rep..

[110] Saville J.T., McDermott B.K., Fletcher J.M., Fuller M. (2019). Disease and Subtype Specific Signatures Enable Precise Diagnosis of the Mucopolysaccharidoses. Genet. Med..

[111] Tomatsu S., Fujii T., Fukushi M., Oguma T., Shimada T., Maeda M., Kida K., Shibata Y., Futatsumori H., Montaño A.M. (2013). Newborn Screening and Diagnosis of Mucopolysaccharidoses. Mol. Genet. Metab..

[112] Pajares S., Arias A., García-Villoria J., Macías-Vidal J., Ros E., de las Heras J., Girós M., Coll M.J., Ribes A. (2015). Cholestane-3β,5α,6β-Triol: High Levels in Niemann-Pick Type C, Cerebrotendinous Xanthomatosis, and Lysosomal Acid Lipase Deficiency. J. Lipid Res..

[113] Boenzi S., Deodato F., Taurisano R., Goffredo B.M., Rizzo C., Dionisi-Vici C. (2016). Evaluation of Plasma Cholestane-3β,5α,6β-Triol and 7-Ketocholesterol in Inherited Disorders Related to Cholesterol Metabolism. J. Lipid Res..

[114] Sidhu R., Kell P., Dietzen D.J., Farhat N.Y., Do A.N.D., Porter F.D., Berry-Kravis E., Reunert J., Marquardt T., Giugliani R. (2020). Application of a Glycinated Bile Acid Biomarker for Diagnosis and Assessment of Response to Treatment in Niemann-Pick Disease Type C1. Mol. Genet. Metab..

[115] Sidhu R., Mondjinou Y., Qian M., Song H., Kumar A.B., Hong X., Hsu F.-F., Dietzen D.J., Yanjanin N.M., Porter F.D. (2019). N-Acyl-O-Phosphocholineserines: Structures of a Novel Class of Lipids That Are Biomarkers for Niemann-Pick C1 Disease. J. Lipid Res..

[116] Maekawa M., Jinnoh I., Matsumoto Y., Narita A., Mashima R., Takahashi H., Iwahori A., Saigusa D., Fujii K., Abe A. (2019). Structural Determination of Lysosphingomyelin-509 and Discovery of Novel Class Lipids from Patients with Niemann-Pick Disease Type C. Int. J. Mol. Sci..

[117] Giese A.-K., Mascher H., Grittner U., Eichler S., Kramp G., Lukas J., te Vruchte D., Al Eisa N., Cortina-Borja M., Porter F.D. (2015). A Novel, Highly Sensitive and Specific Biomarker for Niemann-Pick Type C1 Disease. Orphanet J. Rare Dis..

[118] Maekawa M., Jinnoh I., Narita A., Iida T., Saigusa D., Iwahori A., Nittono H., Okuyama T., Eto Y., Ohno K. (2019). Investigation of Diagnostic Performance of Five Urinary Cholesterol Metabolites for Niemann-Pick Disease Type C. J. Lipid Res..

[119] Maekawa M., Narita A., Jinnoh I., Iida T., Marquardt T., Mengel E., Eto Y., Clayton P.T., Yamaguchi H., Mano N. (2019). Diagnostic Performance Evaluation of Sulfate-Conjugated Cholesterol Metabolites as Urinary Biomarkers of Niemann-Pick Disease Type C. Clin. Chim. Acta.

[120] Porter F.D., Scherrer D.E., Lanier M.H., Langmade S.J., Molugu V., Gale S.E., Olzeski D., Sidhu R., Dietzen D.J., Fu R. (2010). Cholesterol Oxidation Products Are Sensitive and Specific Blood-Based Biomarkers for Niemann-Pick C1 Disease. Sci. Transl. Med..

[121] Jiang X., Sidhu R., Porter F.D., Yanjanin N.M., Speak A.O., te Vruchte D.T., Platt F.M., Fujiwara H., Scherrer D.E., Zhang J. (2011). A Sensitive and Specific LC-MS/MS Method for Rapid Diagnosis of Niemann-Pick C1 Disease from Human Plasma. J. Lipid Res..

[122] Young S.P., Piraud M., Goldstein J.L., Zhang H., Rehder C., Laforet P., Kishnani P.S., Millington D.S., Bashir M.R., Bali D.S. (2012). Assessing Disease Severity in Pompe Disease: The Roles of a Urinary Glucose Tetrasaccharide Biomarker and Imaging Techniques. Am. J. Med. Genet. C Semin Med. Genet..

[123] Potter J.E., Petts G., Ghosh A., White F.J., Kinsella J.L., Hughes S., Roberts J., Hodgkinson A., Brammeier K., Church H. (2021). Enzyme Replacement Therapy and Hematopoietic Stem Cell Transplant: A New Paradigm of Treatment in Wolman Disease. Orphanet J. Rare Dis..

[124] Kannenberg F., Nofer J.-R., Schulte E., Reunert J., Marquardt T., Fobker M. (2017). Determination of Serum Cholestane-3β,5α,6β-Triol by Gas Chromatography-Mass Spectrometry for Identification of Niemann-Pick Type C (NPC) Disease. J. Steroid Biochem. Mol. Biol..

[125] Polo G., Burlina A., Furlan F., Kolamunnage T., Cananzi M., Giordano L., Zaninotto M., Plebani M., Burlina A. (2016). High Level of Oxysterols in Neonatal Cholestasis: A Pitfall in Analysis of Biochemical Markers for Niemann-Pick Type C Disease. Clin. Chem. Lab. Med..

[126] Dang Do A.N., Chang I.J., Jiang X., Wolfe L.A., Ng B.G., Lam C., Schnur R.E., Allis K., Hansikova H., Ondruskova N. (2023). Elevated Oxysterol and N-Palmitoyl-O-Phosphocholineserine Levels in Congenital Disorders of Glycosylation. J. Inherit. Metab. Dis..

[127] Welford R.W.D., Garzotti M., Marques Lourenço C., Mengel E., Marquardt T., Reunert J., Amraoui Y., Kolb S.A., Morand O., Groenen P. (2014). Plasma Lysosphingomyelin Demonstrates Great Potential as a Diagnostic Biomarker for Niemann-Pick Disease Type C in a Retrospective Study. PLoS ONE.

[128] Young S.P., Zhang H., Corzo D., Thurberg B.L., Bali D., Kishnani P.S., Millington D.S. (2009). Long-Term Monitoring of Patients with Infantile-Onset Pompe Disease on Enzyme Replacement Therapy Using a Urinary Glucose Tetrasaccharide Biomarker. Genet. Med..

[129] Piraud M., Pettazzoni M., Lavoie P., Ruet S., Pagan C., Cheillan D., Latour P., Vianey-Saban C., Auray-Blais C., Froissart R. (2018). Contribution of Tandem Mass Spectrometry to the Diagnosis of Lysosomal Storage Disorders. J. Inherit. Metab. Dis..

[130] Hallgren P., Lindberg B.S., Lundblad A. (1977). Quantitation of Some Urinary Oligosaccharides during Pregnancy and Lactation. J. Biol. Chem..

[131] Arvio M., Mononen I. (2016). Aspartylglycosaminuria: A Review. Orphanet J. Rare Dis..

[132] Haijes H.A., de Sain-van der Velden M.G.M., Prinsen H.C.M.T., Willems A.P., van der Ham M., Gerrits J., Couse M.H., Friedman J.M., van Karnebeek C.D.M., Selby K.A. (2019). Aspartylglycosamine Is a Biomarker for NGLY1-CDDG, a Congenital Disorder of Deglycosylation. Mol. Genet. Metab..

[133] van der Ham M., Prinsen B.H.C.M.T., Huijmans J.G.M., Abeling N.G.G.M., Dorland B., Berger R., de Koning T.J., de Sain-van der Velden M.G.M. (2007). Quantification of Free and Total Sialic Acid Excretion by LC-MS/MS. J. Chromatogr. B Anal. Technol. Biomed. Life Sci..

[134] Alroy J., Ucci A.A. (2006). Skin Biopsy: A Useful Tool in the Diagnosis of Lysosomal Storage Diseases. Ultrastruct. Pathol..

[135] Huizing M., Hackbarth M.E., Adams D.R., Wasserstein M., Patterson M.C., Walkley S.U., Gahl W.A. (2021). Free Sialic Acid Storage Disorder: Progress and Promise. Neurosci. Lett..

[136] Dyment D.A., Bennett S.A., Medin J.A., Levade T., Adam M.P., Mirzaa G.M., Pagon R.A., Wallace S.E., Bean L.J., Gripp K.W., Amemiya A. (1993). ASAH1-Related Disorders. GeneReviews^®^.

[137] Hoffman E.P., Barr M.L., Giovanni M.A., Murray M.F., Adam M.P., Mirzaa G.M., Pagon R.A., Wallace S.E., Bean L.J., Gripp K.W., Amemiya A. (1993). Lysosomal Acid Lipase Deficiency. GeneReviews^®^.

[138] Thurberg B.L., Wasserstein M.P., Schiano T., O’Brien F., Richards S., Cox G.F., McGovern M.M. (2012). Liver and Skin Histopathology in Adults with Acid Sphingomyelinase Deficiency (Niemann-Pick Disease Type B). Am. J. Surg. Pathol..

[139] Stepien K.M., Roncaroli F., Turton N., Hendriksz C.J., Roberts M., Heaton R.A., Hargreaves I. (2020). Mechanisms of Mitochondrial Dysfunction in Lysosomal Storage Disorders: A Review. J. Clin. Med..

