# Supplementary material for: Hepatomegaly and Splenomegaly: An Approach to the Diagnosis of Lysosomal Storage Diseases
Source: J Clin Med. 2024 Mar 2;13(5):1465. doi: 10.3390/jcm13051465 (PMC10932313; doi:10.3390/jcm13051465)
Supplement: Supplementary file 1 [file jcm-13-01465-s001.zip › jcm-2882924-supplementary.pdf]

Supplemental materials

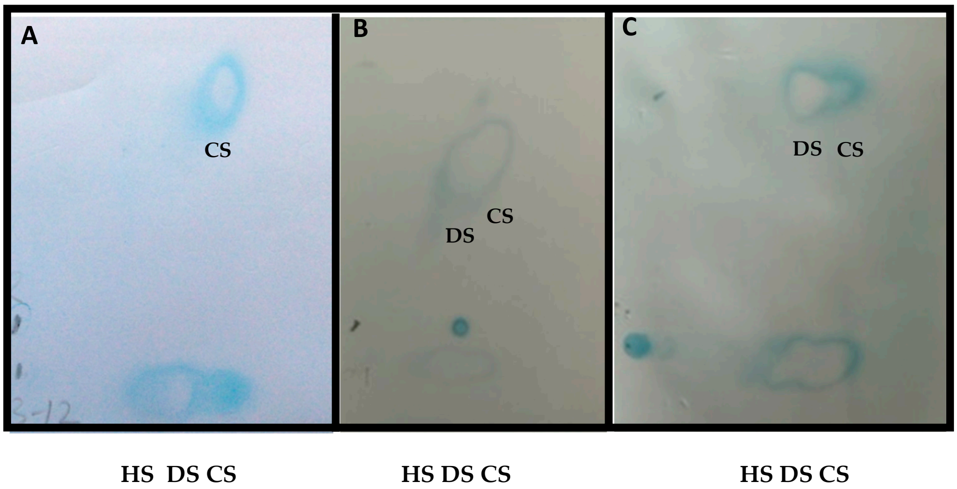

Supplemental Figure S1. Two-dimensional electrophoresis urine GAGs. **A.** Normal, only CS present. **B.** Attenuated Mucopolysaccharidosis type VI, less DS than CS. **C.** Mucopolysaccharidosis type VI, more DS than CS.

CS = Chondroitin sulphate; DS = Dermatan sulphate; GAGs = Glycosaminoglycans; HS = Heparan sulphate

GAGs molecules are net negatively charged and respond to an electrical charge. Each type of molecule responds by a differing amount corresponding to the overall charge of the atoms in the molecule and their orientation with respect to each other. By passing a low-voltage electrical current through a solution of the molecules suspended on an appropriate membrane (cellulose acetate) and in two differing buffer solutions in different directions, a distinctive pattern can be obtained. Mucopolysaccharidoses exhibit a characteristic GAGs excretion pattern which is distinguishable after two-dimensional electrophoresis.

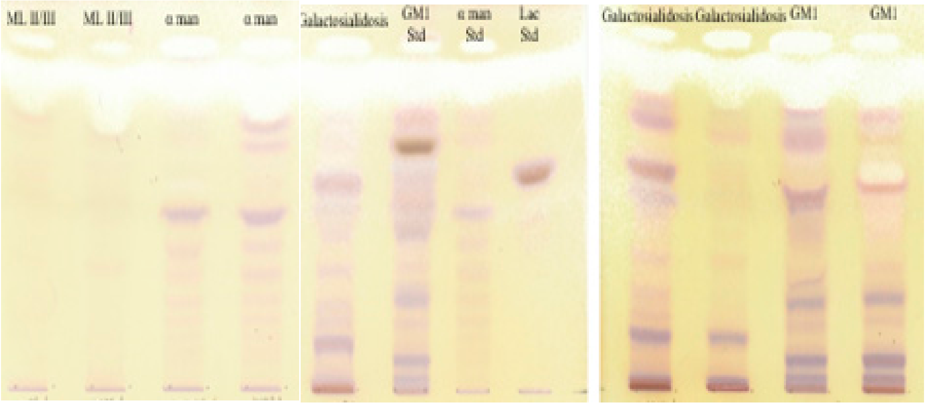

Supplemental Figure S2. Oligosaccharide Thin Layer Chromatography Plate

Examples of Oligosaccharide and Sialic Acid thin layer chromatography plates ran contemporaneously. In assay standards and known affected urine samples applied and run with patient samples each time analysis is performed. Left to right: 2 x Mucolipidosis II/III patient urine (normal oligosaccharide content), 2 x Alpha Mannosidosis patient urine (trisaccharide band and laddering), 1 x Galactosialidosis patient urine (heavy baseline), in assay standards (GM1 gangliosidosis patient urine, Alpha mannosidosis patient urine, lactose), 2 x Galactosialidosis patient urine (heavy baseline), 2 x GM1 gangliosidosis patient urine (heavy bands near baseline).
